# Persistence of SARS-CoV-2 Antibodies in Vaccinated Health Care Workers Analyzed by Coronavirus Antigen Microarray

**DOI:** 10.3389/fimmu.2022.817345

**Published:** 2022-04-12

**Authors:** Sina Hosseinian, Kathleen Powers, Milind Vasudev, Anton M. Palma, Rafael de Assis, Aarti Jain, Peter Horvath, Paramveer S. Birring, Rana Andary, Connie Au, Brandon Chin, Ghali Khalil, Jenny Ventura, Madeleine K. Luu, Cesar Figueroa, Joshua M. Obiero, Emily Silzel, Rie Nakajima, William Thomas Gombrich, Algis Jasinskas, Frank Zaldivar, Sebastian Schubl, Philip L. Felgner, Saahir Khan, The Specimen Collection Group

**Affiliations:** ^1^ School of Medicine, University of California Irvine, Irvine, CA, United States; ^2^ Institute for Clinical and Translational Science, University of California Irvine, Irvine, CA, United States; ^3^ Department of Physiology and Biophysics, University of California Irvine, Irvine, CA, United States; ^4^ School of Biological Sciences, University of California Irvine, Irvine, CA, United States; ^5^ Department of Surgery, School of Medicine, University of California Irvine, Irvine, CA, United States; ^6^ Department of Pediatrics, University of California Irvine, Irvine, CA, United States; ^7^ Department of Medicine, Keck School of Medicine, University of Southern California, Los Angeles, CA, United States

**Keywords:** serology, SARS-CoV-2, healthcare workers, antibodies, microarray, vaccine, mRNA

## Abstract

Recent studies provide conflicting evidence on the persistence of SARS-CoV-2 immunity induced by mRNA vaccines. Here, we aim to quantify the persistence of humoral immunity following vaccination using a coronavirus antigen microarray that includes 10 SARS-CoV-2 antigens. In a prospective longitudinal cohort of 240 healthcare workers, composite SARS-CoV-2 IgG antibody levels did not wane significantly over a 6-month study period. In the subset of the study population previously exposed to SARS-CoV-2 based on seropositivity for nucleocapsid antibodies, higher composite anti-spike IgG levels were measured before the vaccine but no significant difference from unexposed individuals was observed at 6 months. Age, vaccine type, or worker role did not significantly impact composite IgG levels, although non-significant trends towards lower antibody levels in older participants and higher antibody levels with Moderna vaccine were observed at 6 months. A small subset of our cohort were classified as having waning antibody titers at 6 months, and these individuals were less likely to work in patient care roles and more likely to have prior exposure to SARS-CoV-2.

## Introduction

Since the initial 2019 outbreak of the novel beta coronavirus SARS-CoV-2, rapid international spread of the COVID-19 disease has resulted in a global pandemic. In efforts to contain the spread and severity of COVID-19, the FDA approved the emergency distribution of mRNA vaccines BNT162b and mRNA1273 in December of 2020. Both vaccines provide high rates of protective efficacy of up to 95% against the targeted virus strain following two doses administered at least 3-4 weeks apart (1, 2).

Here, we seek to analyze the persistence of SARS-CoV-2 antibody responses induced by 2-dose mRNA vaccines in a health care worker population using a coronavirus antigen microarray. This serological analysis can yield significant insight into comparative antibody responses following vaccination and natural infection. Of particular importance, binding antibodies against SARS-CoV-2 antigens have been shown to correlate strongly with neutralizing antibodies, which are a critical component of clinical immunity (3–6).

In prior studies, subjects who received two doses of mRNA vaccine developed significant levels of IgM and IgG against SARS-CoV-2 spike (S) proteins and receptor-binding domain (RBD) titers (7). Anti-spike protein IgG levels were reported to increase exponentially following initial vaccination but plateau by 21 days. After the second dose, antibody levels increased even further and remained elevated (7, 8). Recent studies provide conflicting evidence on the longitudinal efficacy of the mRNA vaccines- some studies report waning begins as early as 10 weeks (9), others show age (10), vaccine type (11), and prior exposure (11) to be significant factors in the humoral response. Others report waning over the course of 6 months (12, 13), while some report non-waning in both mRNA vaccines and non-mRNA vaccines (14, 15). Here, we evaluate the effect of these factors on humoral immunity up to 6 months following SARS-CoV-2 vaccination.

## Methods

### Study Population

This study was approved by the institutional review board (IRB) of the University of California Irvine (UCI) prior to initiation of the study. Widespread mRNA vaccination of healthcare workers (HCWs) at UC Irvine Health began in December 2020, administering over 16,000 doses of mRNA1273 (Moderna Inc.) or the BNT162b (Pfizer Inc. and BioNTech Inc.) vaccines within the first 4 months. All HCWs working at the UCI Medical Center, located in Orange County CA, were invited to receive serological testing by providing serum blood samples *via* fingerstick at the time of vaccination and follow-up testing at approximately 2 months, 4 months, and 6 months post-final dose of vaccination. All blood samples were brought to the Institute for Clinical and Translational Science Core Laboratory at the UCI Medical Center. Serum samples were centrifuged using the Eppendorf 5415R and spun at 3000xg for 5 minutes. Serum was quickly transferred into a clean sterile tube and frozen at -80°C until analyzed for Igs. Reports of their serological test results were returned within 4 weeks of receiving the test. At each assessment, demographic and work-related characteristics, testing frequency, exposure risk, and symptom history were collected *via* surveys administered prior to serum sample collection. Longitudinal participation was encouraged through an aggressive email campaign as well as ensuring that participants received a report of their antibody titers, but not every subject participated at every time point. A total of 956 HCWs were recruited for longitudinal follow-up. Eligibility for this analysis was restricted to 240 HCWs who provided blood specimens and survey data at multiple time points.

### Coronavirus Antigen Microarray

1,559 independent finger stick blood serum samples were collected over the 6-month period for analysis. Specimens were probed and analyzed on a coronavirus antigen microarray (CoVAM) for IgG and IgM antibodies against 37 antigens from SARS-CoV-2, other coronaviruses, and other respiratory viruses using a coronavirus antigen microarray (**eFigure 1**). The CoVAM contained 10 SARS-CoV-2 antigens including nucleocapsid protein (NP) and several varying fragments of the spike (S) protein, as well as 4 SARS, 3 MERS, 12 Common CoV, and 8 influenza antigens. A full list of antigens used in the assay can be found in **Supplementary Table 1**. Samples were tested in triplicate.

The data analysis was carried out according to the following general pipeline (see Online-Only Methods for details): For each sample, the average reactivity to the printing buffer was subtracted from each spot. The arrays were normalized according to the composite method described elsewhere (16–18). Reactivity assessment was performed using a logistic regression model consisting of a weighted combination of antigens as described elsewhere (18, 19). In summary, a generalized linear model (GLM) was built using 6 antigens (SARS.CoV.2.S1.RBD.mFc, SARS.CoV.2.Spike.RBD.His.HEK, SARS.CoV.2_S1, SARS.CoV.2.NP, SARS.CoV.2 S2, SARS.CoV.2. S1.HisTag). This model was found to be 93% sensitive and 98% specific in correctly classifying 91 PCR-positive cases and 88 pre-pandemic negative control (18, 19). The model was then used to generate a weighted composite measure of IgG reactivity on all titers, with weights corresponding to each antigen’s relative importance in the model. This composite IgG reactivity measure was scaled up to represent the weighted mean fluorescence intensity (MFI) of all antigens assayed in the CoVAM. Here, we utilized a model containing all SARS-CoV-2 antigens as above with the exception of NP, as this antigen was used to classify prior exposure to SARS-CoV-2 in a subgroup analysis.

To determine the relative anti SARS-CoV-2 antibody reactive levels at the last time point, as well as to identify individuals for whom the antibody levels significantly declined, first all individuals with at least two time points were selected. Then, for each individual, the sample with the closest time point to 80 days was identified. All individuals for whom the last time point coincided with the 80 days post vaccination time point were dropped. Lastly, the individuals were classified as having waning antibodies when, the median signal intensity of all SARS-CoV-2 antigens at the final time point was lower than the median of these antigens at the closest to day 80 time point (a p < 0.05, Wilcoxon test, was considered significant). A boxplot of the median antibody reactivity at the last time point, for all selected individuals can be visualized on **Figure 3**. Individual demographics for either the waning or non-waning groups obtained from the consent form are listed on **Table 3**.

### Statistical Analysis

In order to characterize SARS-CoV-2 antibody response over time, we fit a linear mixed effect model of the composite IgG reactivity measure using all available data from the n=240 HCWs with at least 2 time points available. Due to the variability in the timing of the tests across individuals, we report the model-estimated composite IgG reactivity means and 95% confidence intervals (CI) at pre-vaccine, 2 months, 4 months and 6 months post-first dose, and compared the changes over time. We then explored differences in long-term antibody response by individual characteristics hypothesized to influence the magnitude and durability of the vaccine-induced antibody response: sex [male or female, by self-report], age [≥55 vs. <55 years, by self-report], HCW role [patient care vs. non-patient care role], race [Asian, Latino, White, Other, by self-report], presence of obesity and/or diabetes, hypertension, vaccine type [mRNA1273 vs. BNT162b], and prior COVID-19 exposure [defined by presence of SARS-CoV-2 NP antibody reactivity at baseline]. We tested each potential moderator individually by fitting the same linear mixed effect model with the inclusion of an interaction term between that variable and time (e.g., time * age≥55 vs. <55 years). All analyses were conducted using R v4.1.1.

## Results

### SARS-CoV-2 Antibody Levels Persist 6 Months Post-Vaccination

**Figure 1:**
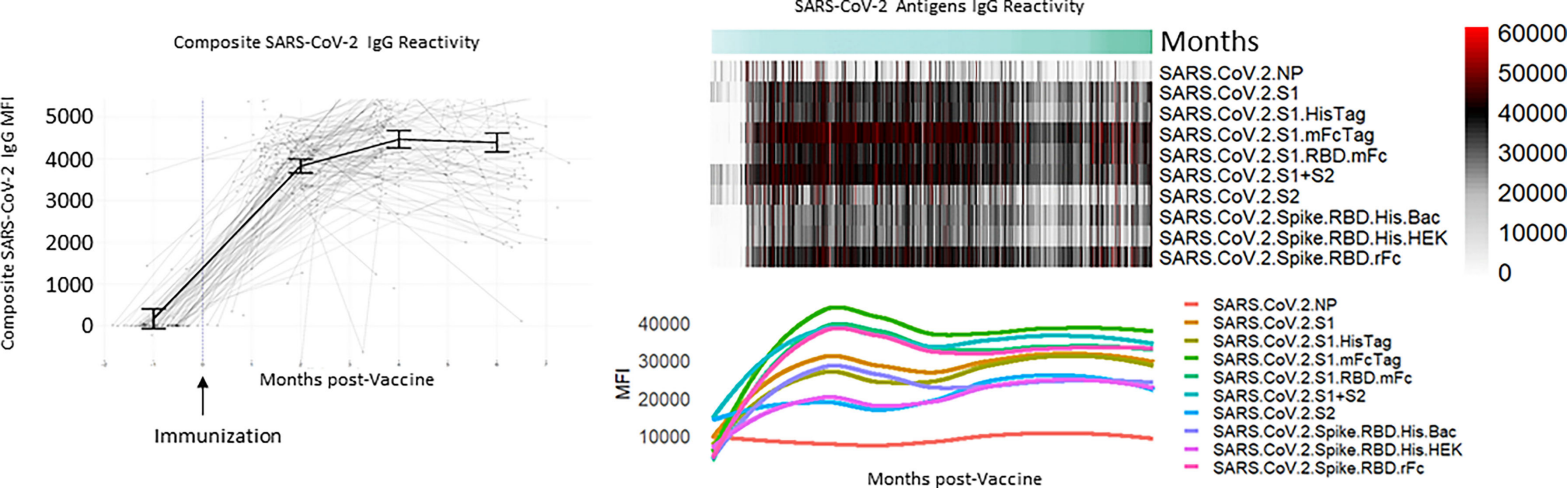
Composite and individual SARS-CoV-2 IgG levels over time for the longitudinal cohort. Background lines (left) representing individual study participants and thick solid line representing mean antibody level at baseline, 2 months, 4 months, and 6 months with error bars representing 95% confidence intervals with heatmap and individual antibody plots.

**Figure 2:**
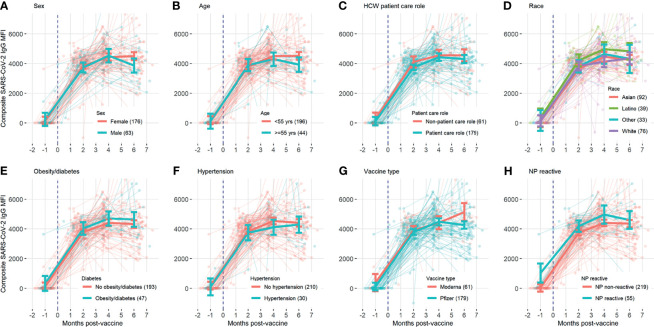
Composite SARS-CoV-2 IgG levels over time, with background lines representing individual study participants and thick solid line representing mean antibody level at baseline, 2 months, 4 months, and 6 months with error bars representing 95% confidence intervals, compared for subgroups divided by **(A)** sex, **(B)** age, **(C)** HCW patient care role, **(D)** race, **(E)** obesity/diabetes, **(F)** hypertension, **(G)** vaccine type, and **(H)** previous exposure.

A total of 629 tests were collected from 240 HCWs who provided at least 2 samples (mean number of samples 2.6, range 2-6, **Table 1**). Antibody responses significantly increased in the 2 months following vaccination (mean [95% CI] composite IgG MFI: baseline: 175 [-63, 308], 2mo: 3829 [3665, 3993]; baseline to 2 months, p<0.001) and increased further at 4 months (mean [95% CI] composite IgG MFI: 4mo: 4471 [4263, 4679]; 2 to 4 months, p<0.001). Antibody levels plateaued at the 4-month timepoint; notably, we observed no evidence of significant waning from 4 to 6 months (p=0.959, **Figure 1** and **Table 2**). Comparisons by sex did not show any significant difference, although men did show a trend towards lower composite IgGs at 6 months (men vs. women at 6 months: p=0.301, **Figure 2A**). Comparisons by age showed modest waning from 4 to 6 months in the older age group who were ≥55 years; however, these differences were not statistically significant (4 to 6 months among men: p=0.949, **Figure 2B** and **Table 2**). No statistically significant differences in antibody levels were observed between participants segregated by patient care role, race, presence of obesity and/or diabetes, or affliction of hypertension (**Figures 2C–F** and **Table 2**). Similarly, no statistically significant differences in antibody levels were observed between those who received the BNT162b or mRNA1273 vaccines, although there was a non-significant trend towards higher antibody levels at 6 months with mRNA1273 (**Figure 2G**). HCWs who had evidence of prior exposure to COVID-19 as defined by NP reactivity at baseline haxd higher composite IgG levels at baseline but did not differ significantly at post-vaccine time points up to 6 months; furthermore, their antibody levels increased significantly post-vaccination (**Figure 2H**). With respect to individual antigens, vaccine-induced antibodies were directed primarily against the S1 and RBD domains of spike protein, to a lesser extent against the S2 domain, and not at all against the nucleocapsid protein as expected based on design of mRNA vaccines (**eFigures 2**–**4**).

**Table 1:** Characteristics of study participants compared between all study participants and the longitudinal cohort who provided data at multiple time points post vaccination.

	HCWs (n=240)
No. tests	
2	152 (63%)
3	53 (22%)
4	21 (9%)
5	8 (3%)
6	6 (3%)
Gender	
Female	176 (73%)
Male	63 (26%)
Non-binary	1 (0%)
Age (years)	
20-29	52 (22%)
30-39	77 (32%)
40-49	52 (22%)
50-59	40 (17%)
60-69	20 (8%)
70+	2 (1%)
Race	
Asian	92 (38%)
White	76 (32%)
Latino	39 (16%)
Other	33 (14%)
Role	
Administrative	27 (11%)
Clinical staff	31 (13%)
Food/EVS	9 (4%)
Nurse	100 (42%)
Physician	26 (11%)
Student	22 (9%)
Other	25 (10%)
Obesity/diabetes	
BMI >30 or diabetes	47 (20%)
Neither	193 (80%)
Hypertension	
Yes	30 (13%)
No	210 (88%)
History of tobacco use	
Yes	3 (1%)
No	237 (99%)
NP reactive at baseline	
Reactive	41 (17%)
Non-reactive	199 (83%)

**Table 2:** Composite SARS-CoV-2 IgG levels compared between time points for subgroups of study participants divided by gender, age, race, occupation role, and presence of co-morbidities.

Variable	Category	N	Composite SARS-CoV-2 IgG MFI, mean (95% CI)^a^					p-value^b^		
			Pre-vaccine	2mo	4mo	6mo		Pre vs. 2mo	2mo vs. 4mo	4mo vs. 6mo
Overall		240	175 (0, 408)	3829 (3665, 3993)	4471 (4263, 4679)	4396 (4166, 4625)		<0.001*	<0.001*	*0.959*
Sex^c^	Female	176	146 (0, 424)	3856 (3669, 4043)	4450 (4217, 4682)	4506 (4243, 4770)		<0.001*	0.001*	*0.999*
	Male	63	250 (0, 688)	3744 (3418, 4071)	4541 (4087, 4995)	3878 (3407, 4348)		<0.001*	*0.077*	*0.430*
	*Female vs. male*		*0.999*	*0.999*	*0.999*	*0.301*				
Age	<55 years	196	187 (0, 453)	3814 (3635, 3992)	4515 (4282, 4748)	4524 (4265, 4784)		<0.001*	<0.001*	*0.999*
	≥55 years	44	122 (0, 631)	3908 (3499, 4316)	4305 (3852, 4759)	3939 (3448, 4429)		<0.001*	*0.877*	*0.949*
	*<55 years vs. ≥55 years*		*0.999*	*0.999*	*0.993*	*0.434*				
Race	Asian	92	112 (0, 505)	3654 (3395, 3912)	4463 (4133, 4793)	4250 (3906, 4595)		<0.001*	0.009*	*0.999*
	White	76	141 (0, 518)	3846 (3544, 4149)	4136 (3768, 4503)	4365 (3969, 4762)		<0.001*	*0.997*	*0.999*
	Latino	39	369 (0, 990)	4223 (3817, 4629)	4978 (4499, 5458)	4834 (4275, 5392)		<0.001*	*0.501*	*0.999*
	Other	33	167 (0, 869)	3824 (3406, 4242)	4650 (4004, 5295)	4327 (3379, 5274)		<0.001*	*0.709*	*0.999*
	*By race*		*0.999*	*0.999*	<0.001*	<0.001*				
NP reactivity	Non-reactive	199	26 (0, 191)	3712 (3595, 3828)	4413 (4196, 4630)	4362 (4118, 4606)		<0.001*	<0.001*	*0.999*
	Reactive	41	1093 (671, 1514)	4211 (3958, 4464)	4972 (4352, 5592)	4610 (4012, 5208)		<0.001*	*0.388*	*0.991*
	*Non-reactive vs. reactive*		*0.084*	*0.435*	*0.703*	*0.995*				
Patient care	Patient care role	179	122 (-162, 407)	3755 (3573, 3938)	4436 (4186, 4685)	4320 (4041, 4599)		<0.001*	<0.001*	*0.998*
	Non-patient care role	61	285 (-134, 705)	4115 (3747, 4484)	4563 (4189, 4937)	4560 (4157, 4964)		<0.001*	*0.658*	*0.999*
	*By patient care role*		*0.998*	*0.674*	*0.999*	*0.979*				
Hypertension	No hypertension	210	184 (-75, 444)	3839 (3666, 4012)	4543 (4315, 4771)	4415 (4161, 4669)		<0.001*	<0.001*	*0.995*
	Hypertension	30	102 (-465, 669)	3747 (3233, 4261)	4117 (3610, 4623)	4293 (3749, 4838)		<0.001*	*0.964*	*0.999*
	*No hypertension vs. hypertension*		*0.999*	*0.999*	*0.804*	*0.999*				
Obesity/diabetes	No obesity and diabetes	193	127 (-140, 394)	3791 (3613, 3968)	4419 (4189, 4649)	4329 (4070, 4588)		<0.001*	<0.001*	*0.999*
	Obesity or diabetes	47	335 (-163, 833)	4029 (3603, 4455)	4710 (4227, 5194)	4646 (4148, 5145)		<0.001*	*0.378*	*0.999*
	*Any vs. none*		*0.996*	*0.972*	*0.962*	*0.954*				

a Composite SARS-CoV-2 IgG MFI mean and 95% confidence interval bounds are post-estimated values calculated based on the fitted regression model.

b p-values indicate strength of evidence for change in Composite SARS-CoV-2 IgG MFI between timepoints at 2 months intervals, against a null hypothesis of no change, as estimated from Wald test on model post-estimated marginal means within each group and timepoint. Asterisks denote significant differences at p<0.05.

c One participant self-reported as non-binary sex and was excluded for the sex-specific analysis.

### SARS-CoV-2 Antibody Waning in Select Individuals

**Figure 3:**
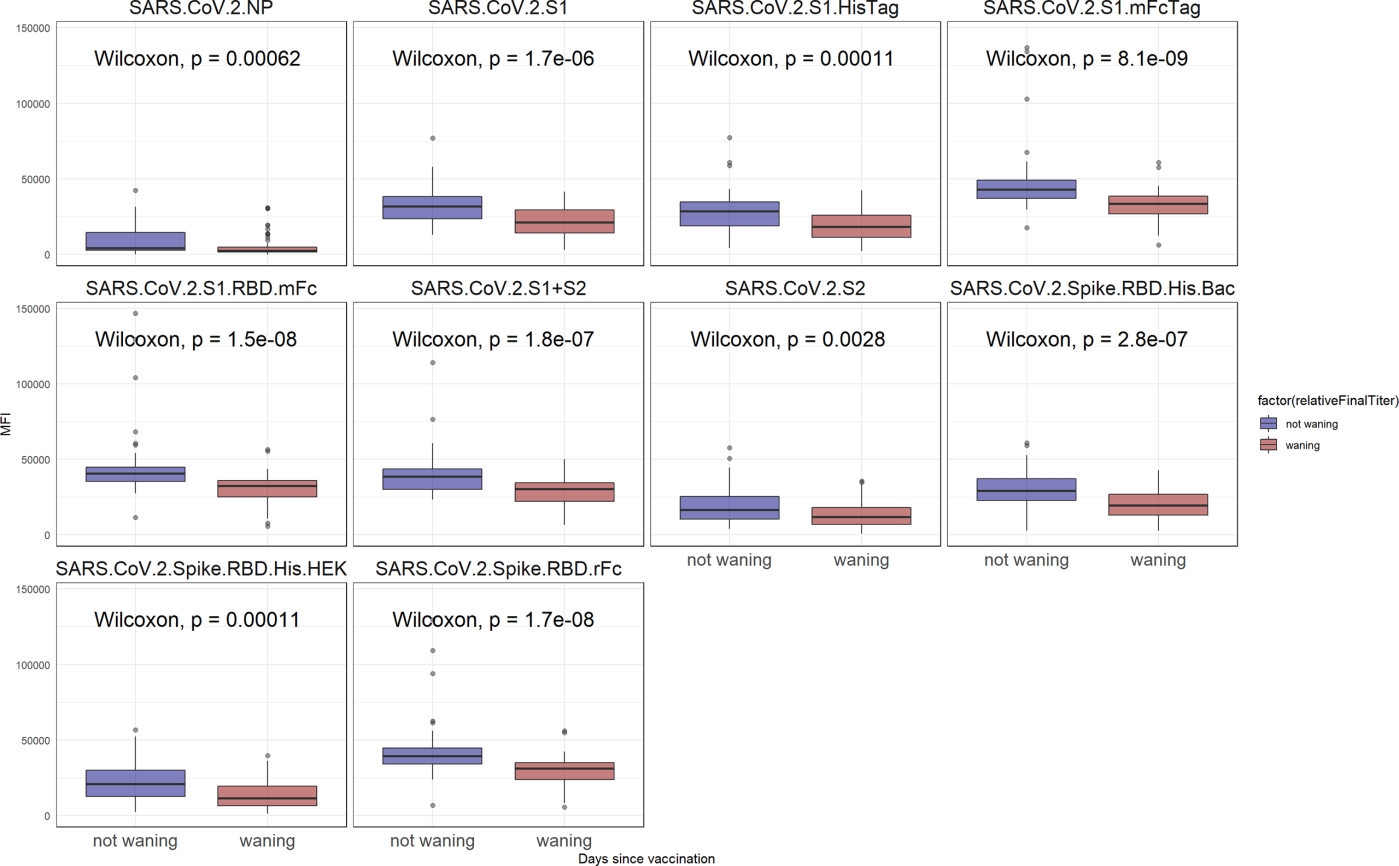
SARS-CoV-2 reactivity after 6 months. In blue, are individuals for whom the reactivity did not significantly wane when compared to the day 80 time point. In red are samples for whom the reactivity has declined (p < 0.05).

Although the overall reactivity did not seem to significantly wane over the observed 6-month period, as seen on **Figure 3**, a subset of study participants could be classified as having waning antibodies against SARS-CoV-2 antigens. A significant difference between individuals with waning antibodies and non-waning antibodies was identified for all SARS-CoV-2 antigens (p < 0.05). The observed reactivity differences are more pronounced for S1-containing antigens, although a significant difference was observed for S2 and NP antigens as well. Among 41 individuals being classified as non-waning and 58 as waning, there was no significant difference between the groups when examining age, gender, vaccine type, time since vaccination, or race (**Table 3**). However, non-waning individuals were less likely to work in patient care roles and were more likely to have presence of NP antibodies.

**Table 3:** Demographic variables in patients with and without waning antibody titers.

	Non-waning (n=41)	Waning (n=58)	p-value
Age	45.15	44.9	0.337
Gender			
Male	9 (21.9)	13 (22.4)	0.969
Female	31 (75.6)	44 (75.9)	
Non-binary	1 (2.4)	1 (1.7)	
Vaccine type			0.985
Moderna	5 (12.2)	7 (12.1)	
Pfizer	36 (87.8)	51 (87.9)	
Race			0.127
Asian	10 (24.4)	30 (51.7)	
White	21 (51.2)	17 (29.3)	
Latino	8 (19.5)	6 (10.3)	
Black	0 (0)	1 (1.7)	
Other	2 (4.9)	4 (6.9)	
Role			0.01*
Patient care	20 (48.8)	43 (74.1)	
Non-patient care	21 (51.2)	15 (25.9)	
NP Antigen			0.03*
Negative	29	51	
Positive	12	7	
Days since vaccine	168.41	176.91	0.262

Demographic data was obtained from survey questions on the consent form. Means are reported with percentages being reported in parenthesis. Values with an asterisk are statically significant with a p value <0.05.

## Discussion

This study utilized a novel immunoassay against 10 different SARS-CoV-2 antigens to measure compositive anti-SARS-CoV-2 IgG levels amongst healthcare workers following vaccination. The test performance of SARS-CoV-2 immunoassays can vary, with more antigens correlating with a higher specificity (20, 21). The CoVAM utilizes multiple antigens to achieve test performance that compares favorably to commercially available assays (18, 22).

Using the CoVAM, we initially observed no significant decline of SARS-CoV-2 antibody levels up to 6 months after the first dose of mRNA vaccine for the general HCW population. Some prior studies show waning of SARS-CoV-2 antibody levels as soon as 10 weeks (9) or 3 months (23) following first dose of mRNA vaccine. We propose that these differences may be due to the use of more antigens in the immunoassay, which is able to detect a broader repertoire of antibodies, and the generalized linear model used to determine composite antibody level, which is able to increase the weight of antibodies specific to SARS-CoV-2. Alternatively, given the high inter-individual variability in antibody levels at all time points, it is possible that this study was underpowered to detect small differences in the antibody response across time points.

Collection of serology samples from patients who had received either the mRNA1273 or the BNT162b mRNA vaccine allowed evaluation of the relative differences in longitudinal antibody levels induced by the two vaccines. Other reports suggested that mRNA1273 may be more effective at sustaining antibody titers long-term (11). No statistically significant differences were observed in antibody responses to the two vaccines at any point time; however, at 6 months post-vaccination, subjects who received the mRNA1273 vaccine showed a non-significant trend towards higher antibody levels compared to those who received the BNT162b vaccine. A follow-up study with a larger sample size may be able to elicit whether this suggested difference is significant.

Although our overall analysis shows no significant decline in 6 months for the general HCW population, a further retrospective analysis identifies a small subset of individuals whose antibodies do in fact wane over the course of the 6 months as compared to the overall cohort. No significant difference was found between the two groups when investigating for age, gender, vaccine type, or race, but surprisingly, waning antibodies were correlated with being involved with a direct patient-care role, being defined as either a nurse, physician, student, or patient care technician. We initially hypothesized that being involved with a patient care role would result in higher, non-waning antibodies, but that is not the case in this study. It is important to note that healthcare workers not involved with patient care were still in contact with patients. In addition, the presence of the NP antibody, which is a marker for prior natural infection with SARS-CoV-2, was higher among non-waning individuals which may be driving the increased persistence of the humoral immune response in these individuals. We hypothesize that workers not involved in patient care may have had less personal protective equipment usage than patient care workers, resulting in a higher prevalence of NP antibody among non-care positions and thus having a higher, non-waning humoral response to the vaccine.

Nearly all immunocompetent individuals develop a humoral immune response following SARS-CoV-2 exposure (24–26). A fraction of our study cohort (20% of the total cohort) included subjects that had received the vaccine after previous exposure to SARS-CoV-2, assessed by presence of antibodies against nucleocapsid protein, which are only found in individuals previously exposed to the virus and not vaccinated individuals. The composite IgG antibody levels were compared between the baseline NP reactive and NP non-reactive participants before and after vaccination. Participants with previous exposure to SARS-CoV-2 had higher IgG antibody levels pre-vaccine (excluding anti-NP antibodies) than participants without prior exposure. The differences in antibody levels between these groups decreased over time and were not statistically significant at any time point following vaccination. Prior studies that report differences in vaccine-induced antibody responses based on prior SARS-CoV-2 exposure focused on shorter time points at 6 to 10 weeks post vaccination (11), it is possible that with a larger sample size, this study would have detected a small difference at early post-vaccine time points, but the observed trends suggest that such a difference does not persist at later post-vaccine time points. Surprisingly, our outreach campaign revealed that many HCWs refused the vaccine due to their prior exposure, believing erroneously that prior exposure is just as effective at eliciting a humoral immune response as vaccination. Our data indicates that even in participants previously exposed to SARS-CoV-2, mRNA vaccination induces a significant increase in humoral immunity, and vaccination produces higher levels of SARS-CoV-2 antibodies compared to prior exposure, corroborating other studies that have found previously infected but unvaccinated individuals to be at a higher risk for contracting severe disease compared to vaccinated individuals (27, 28).

We hypothesized that different subgroups of HCWs would have varying SARS-CoV-2 antibody levels following vaccination, with older individuals having decreasing antibody levels at later time points as observed in prior studies (10, 29, 30). While we observed a trend towards decreasing antibody levels at 6 months post vaccination in participants above the age of 55, this difference was not statistically significant. While other studies examined ages above 65, our study included very few healthcare workers older than 65, so it is possible that the lower age threshold and limited sample size resulted in insufficient power to detect age-related antibody waning. We also stratified healthcare workers by role, hypothesizing that those in patient care roles may have higher antibody levels throughout the 6-month period due to potential exposure to patients with COVID-19. Our data did not show any significant evidence to suggest that patient care role influences antibody levels in the healthcare worker population, suggesting that current approaches to infection prevention among staff in healthcare facilities are effective.

This study is particularly relevant to defining the optimal timing and target populations for additional doses of mRNA vaccine beyond the initial 2-dose series in order to sustain long-term immunity to SARS-CoV-2. Among a cohort of generally immunocompetent healthcare workers, there were some individuals whose antibody levels significantly waned over the course of 6 months. These individuals would likely benefit the most from early administration of additional doses of mRNA vaccine, but further studies are needed to define the optimal approach to identify these individuals with waning antibody levels and to characterize the magnitude of differences in antibody levels that correlate with reduction in clinical immunity.

## Data Availability Statement

The datasets presented in this study can be found in online repositories. The names of the repository/repositories and accession number(s) can be found below: GEO Data Repository, GSE199668.

## Ethics Statement

The studies involving human participants were reviewed and approved by Institutional Review Board of University of California Irvine. The patients/participants provided their written informed consent to participate in this study.

## Specimen Collection Group

Ariana Naaseh, Ava Runge, Shannon Skochko, Steven Tohmasi, Olivia Tsai, Justine Chinn, Jessica Colin Escobar, Christina Grabar, Amanda Leung, and Fjolla Muqolli.

## Author Contributions

SH, KP, MV, PB, RA, CA, BC, GK, JV, MK, SS, PF, and SK conceived and designed research. SH, KP, MV, PB, RA, CA, BC, GK, JV, and ML collected samples. PH and FZ prepared and stored samples. PF and SK designed the microarray. RDA, AaJ, JO, ES, RN, AlJ, and WG constructed the microarray and probed samples. AP and RA analyzed data. SH, KP, MV, AP, RDA, SS, PF, and SK interpreted results of data. AP and RA prepared figures. SH, KP, MV, AP, RDA, PB, ML, and SK drafted the manuscript. SH, KP, MV, AP, RA, FZ, and SK edited and revised manuscript. SH, SS, PF, and SK obtained funding for the project. All authors approved final version of the manuscript.

## Funding

This work was supported by two intramural research grants from the COVID-19 Basic, Translational, and Clinical Research Fund of the University of California Irvine and by the Emergency COVID-19 Research Seed Funding Opportunity from the University of California Office of the President [research grants R00RG2646, R01RG3745]. This work was supported by a grant from the Surgical Infection Society Foundation. Research reported in this publication was supported by The Institute for Clinical and Translational Science of the National Institutes of Health under award number T35DK128788. SK was supported by the National Center for Research Resources and the National Center for Advancing Translational Sciences of the National Institutes of Health [grant KL2 TR001416]. The project described was supported by the National Center for Research Resources and the National Center for Advancing Translational Sciences of the National Institutes of Health [grant UL1 TR001414]. The initial design and construction of the CoVAM was supported by the Prometheus-UMD contract sponsored by the Defense Advanced Research Projects Agency (DARPA) BTO under the auspices of Col. Matthew Hepburn [agreements N66001-17-2-4023, N66001-18-2-4015]. The findings and conclusions in this report are those of the authors and do not necessarily represent the official position or policy of the funding agencies and no official endorsements should be inferred.

## Author Disclaimer

The findings and conclusions in this report are those of the authors and do not necessarily represent the official position or policy of the University of California. The content is solely the responsibility of the authors and does not necessarily represent the official views of the NIH.

## Conflict of Interest

The coronavirus antigen microarray is intellectual property of the Regents of the University of California that is licensed for commercialization to Nanommune Inc. (Irvine, CA), a private company for which PF is the largest shareholder and several co-authors (RA, AaJ, RN, and SK) also own shares. Nanommune Inc. has a business partnership with Sino Biological Inc. (Beijing, China) which expressed and purified the antigens used in this study. KP is invested in mutual funds that have either Pfizer or Moderna holdings: American Funds Fundamental, Federated Hermes Kaufman Fund, and Fidelity Biotechnology Fund.

The remaining authors declare that the research was conducted in the absence of any commercial or financial relationships that could be construed as a potential conflict of interest.

## Publisher’s Note

All claims expressed in this article are solely those of the authors and do not necessarily represent those of their affiliated organizations, or those of the publisher, the editors and the reviewers. Any product that may be evaluated in this article, or claim that may be made by its manufacturer, is not guaranteed or endorsed by the publisher.

## Supplementary Material

Supplementary Table 1 List of antigens used in SARS-CoV-2 microarray.

Supplementary Figure 1 Content of coronavirus antigen microarray with representative microarray images for different time points.

Supplementary Figure 2 Antigen-specific SARS-CoV-2 IgG levels over time, with background lines representing individual study participants and thick solid line representing mean antibody level at baseline, 2 months, 4 months, and 6 months with error bars representing 95% confidence intervals, for **(A)** NP, **(B)** S1+S2, and **(C)** S2 antigens.

Supplementary Figure 3 Antigen-specific SARS-CoV-2 IgG levels over time, with background lines representing individual study participants and thick solid line representing mean antibody level at baseline, 2 months, 4 months, and 6 months with error bars representing 95% confidence intervals, for **(A)** S1, **(B)** S1 with His tag, and **(C)** S1 with mouse Fc tag antigens.

Supplementary Figure 4 Antigen-specific SARS-CoV-2 IgG levels over time, with background lines representing individual study participants and thick solid line representing mean antibody level at baseline, 2 months, 4 months, and 6 months with error bars representing 95% confidence intervals, for **(A)** RBD with His tag produced in HEK-293 cells, **(B)** RBD with rabbit Fc tag, **(C)** RBD with mouse Fc tag, and d) RBD with His tag produced in baculovirus antigens.

Supplementary Figure 5 SARS-CoV-2 reactivity after 6 months. Blue lines indicate individuals for whom reactivity did not significantly wane when compared to the last time point recorded, in red are samples for whom the reactivity has declined (P < 0.05).
