# Supplementary material for: Persistence of SARS-CoV-2 Antibodies in Vaccinated Health Care Workers Analyzed by Coronavirus Antigen Microarray
Source: Front Immunol. 2022 Apr 12;13:817345. doi: 10.3389/fimmu.2022.817345 (PMC9040070; doi:10.3389/fimmu.2022.817345)
Supplement: Supplementary Table 1 — List of antigens used in SARS-CoV-2 microarray. [file DataSheet_1.docx]

| **Antigen ID** | **Virus** | **Antigen** | **Catalog** |
| --- | --- | --- | --- |
| SARS.CoV.2.NP | SARS-CoV-2 | SARS-CoV-2_NP | 40588-V08B |
| SARS.CoV.2.S1+S2 | SARS-CoV-2 | 2019-nCoV Spike Protein(S1+S2 ECD, His tag) | 40589-V08B1 |
| SARS.CoV.2.S2 | SARS-CoV-2 | 2019-nCoV Spike Protein (S2 ECD, His tag) | 40590-V08B |
| SARS.CoV.2.S1.mFcTag | SARS-CoV-2 | 2019-nCoV Spike Protein S1 (mFc Tag) | 40591-V05H1 |
| SARS.CoV.2.S1 | SARS-CoV-2 | SARS-CoV-2_S1(His) | 40591-V08B1 |
| SARS.CoV.2.S1.HisTag | SARS-CoV-2 | 2019-nCoV Spike Protein S1 (His Tag) | 40591-V08H |
| SARS.CoV.2.S1.RBD.mFc | SARS-CoV-2 | SARS-CoV-2 (2019-nCoV) Spike RBD-mFc Recombinant Protein (HPLC-verified) | 40592-V05H |
| SARS.CoV.2.Spike.RBD.His.Bac | SARS-CoV-2 | SARS-CoV-2 (2019-nCoV) Spike RBD-His Recombinant Protein, Baculovirus-Insect Cells | 40592-V08B |
| SARS.CoV.2.Spike.RBD.His.HEK | SARS-CoV-2 | SARS-CoV-2 (2019-nCoV) Spike RBD-His Recombinant Protein, HEK293 | 40592-V08H |
| SARS.CoV.2.Spike.RBD.rFc | SARS-CoV-2 | SARS-CoV-2 (2019-nCoV) Spike RBD-rFc Recombinant Protein | 40592-V31H |
| MERS.CoV.NP | MERS | MERS-CoV (NCoV / Novel coronavirus) Nucleoprotein / NP protein (His Tag) | 40068-V08B |
| MERS.CoV.S1.ECD.1-1297.HisTag | MERS | MERS-CoV (NCoV / Novel coronavirus) Spike Protein (ECD, aa 1-1297, His Tag) | 40069-V08B |
| MERS.CoV.S1.RBD.367.606.rFcTag | MERS | MERS-CoV_S1-RBD,N-(AA367-606,rFcTag) | 40071-V31B1 |
| SARS.CoV.NP | SARS | SARS-CoV_NP(His) | 40143-V08B |
| SARS.CoV.S1.HisTag | SARS | SARS-CoV_S1,(HisTag) | 40150-V08B1 |
| SARS.CoV.S1.RBD.HisTag | SARS | SARS-CoV_S1-RBD,(HisTag) | 40150-V08B2 |
| SARS.CoV.S1.RBD.rFcTag | SARS | SARS-CoV_S1-RBD,rFcTag | 40150-V31B2 |
| hCoV.NL63.S1 | Common Cold CoV | HCoV-NL63_S1 | 40600-V08H |
| hCoV.229E.S1 | Common Cold CoV | HCoV-229E_S1 | 40601-V08H |
| hCoV.OC43.HE | Common Cold CoV | HCoV-OC43_HE | 40603-V08H |
| hCoV.NL63.S1_S2 | Common Cold CoV | HCoV-NL63_S1+S2 | 40604-V08B |
| hCoV.229E.S1_S2 | Common Cold CoV | HCoV-229E_S1+S2 | 40605-V08B |
| hCoV.HKU1.S1_S2 | Common Cold CoV | HCoV-HKU1_S1+S2 | 40606-V08B |
| hCoV.OC43.S1_S2ECD.HisTag | Common Cold CoV | HCoV-OC43_S1+S2 ECD, (His Tag) | 40607-V08B |
| hCoV.NL63.NP | Common Cold CoV | HCoV-NL63_NP, (His Tag) | 40641-V07E |
| hCoV.HKU1.NP | Common Cold CoV | HCoV-HKU1_NP, (His Tag) | 40642-V07E |
| hCoV.OC43.NP | Common Cold CoV | HCoV-OC43_Hemagglutinin esterase Protein (His Tag) | 40643-V07E |
| DcCoV.HKU23.NP | Common Cold CoV | DcCoV_HKU23-368F_NP | 40458-V08B |
| hCoV.HKU1.S1_AA1.760 | Common Cold CoV | HCoV-HKU1_S1,N-(AA1-760) | 40021-V08H |
| Flu.B_Mal/.HA1+HA2 | Influenza | B_B/Malaysia/2506/2004_HA1+HA2 | 11716-V08H |
| Flu.B_Mal/.HA1 | Influenza | B_B/Malaysia/2506/2004_HA1 | 11716-V08H1 |
| Flu.H1N1.HA1+HA2 | Influenza | H1N1_A/Beijing/22808/2009_HA1+HA2 | 40035-V08H |
| Flu.H1N1.HA1 | Influenza | H1N1_A/Beijing/22808/2009_HA1 | 40035-V08H1 |
| Flu.H3N2.HA1+HA2 | Influenza | H3N2_A/Texas/50/2012_HA1+HA2 | 40354-V08B |
| Flu.H3N2.HA1 | Influenza | H3N2_A/Texas/50/2012_HA1 | 40354-V08H1 |
| Flu.B_Phu/.HA1+HA2 | Influenza | B_B/Phuket/3073/2013_HA1+HA2 | 40498-V08B |
| Flu.B_Phu/.HA1 | Influenza | B_B/Phuket/3073/2013_HA1 | 40498-V08H1 |
|  |  |  |  |
