# Supplementary figures and images for: Persistence of SARS-CoV-2 Antibodies in Vaccinated Health Care Workers Analyzed by Coronavirus Antigen Microarray
Source: Front Immunol. 2022 Apr 12;13:817345. doi: 10.3389/fimmu.2022.817345 (PMC9040070; doi:10.3389/fimmu.2022.817345)

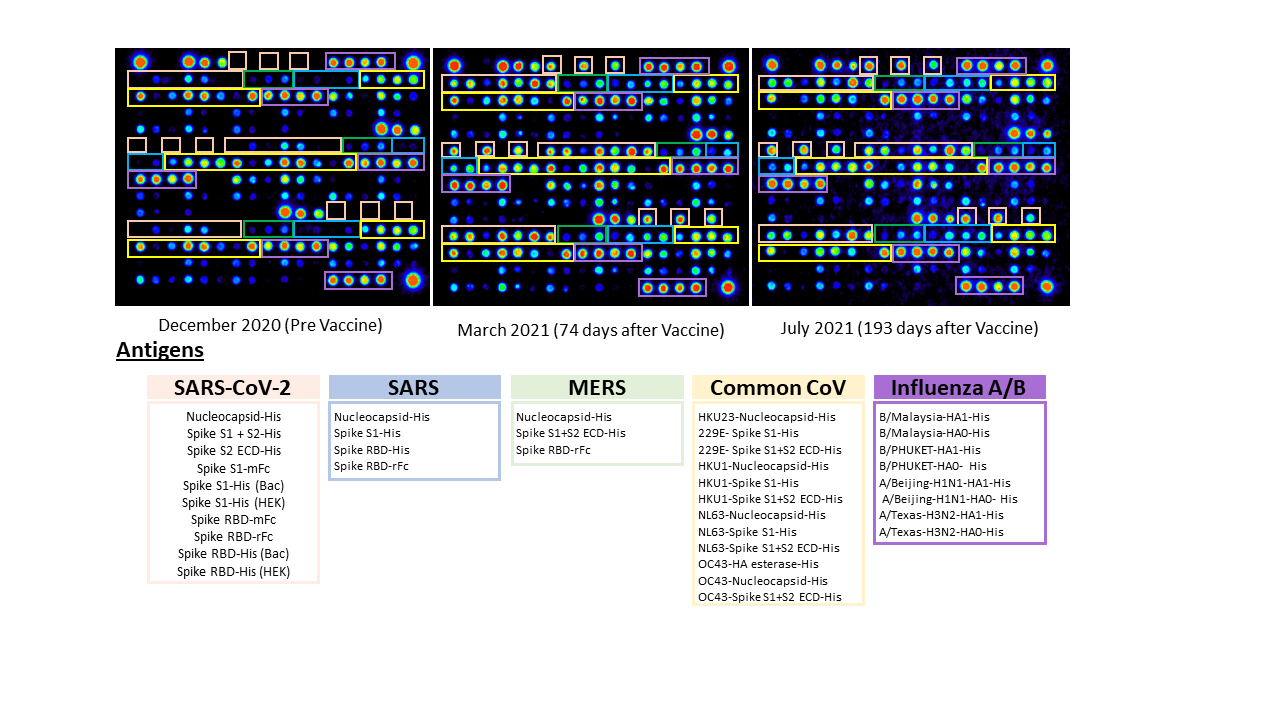

Supplement: Supplementary Figure 1 — Content of coronavirus antigen microarray with representative microarray images for different time points. [file Image_1.tif]

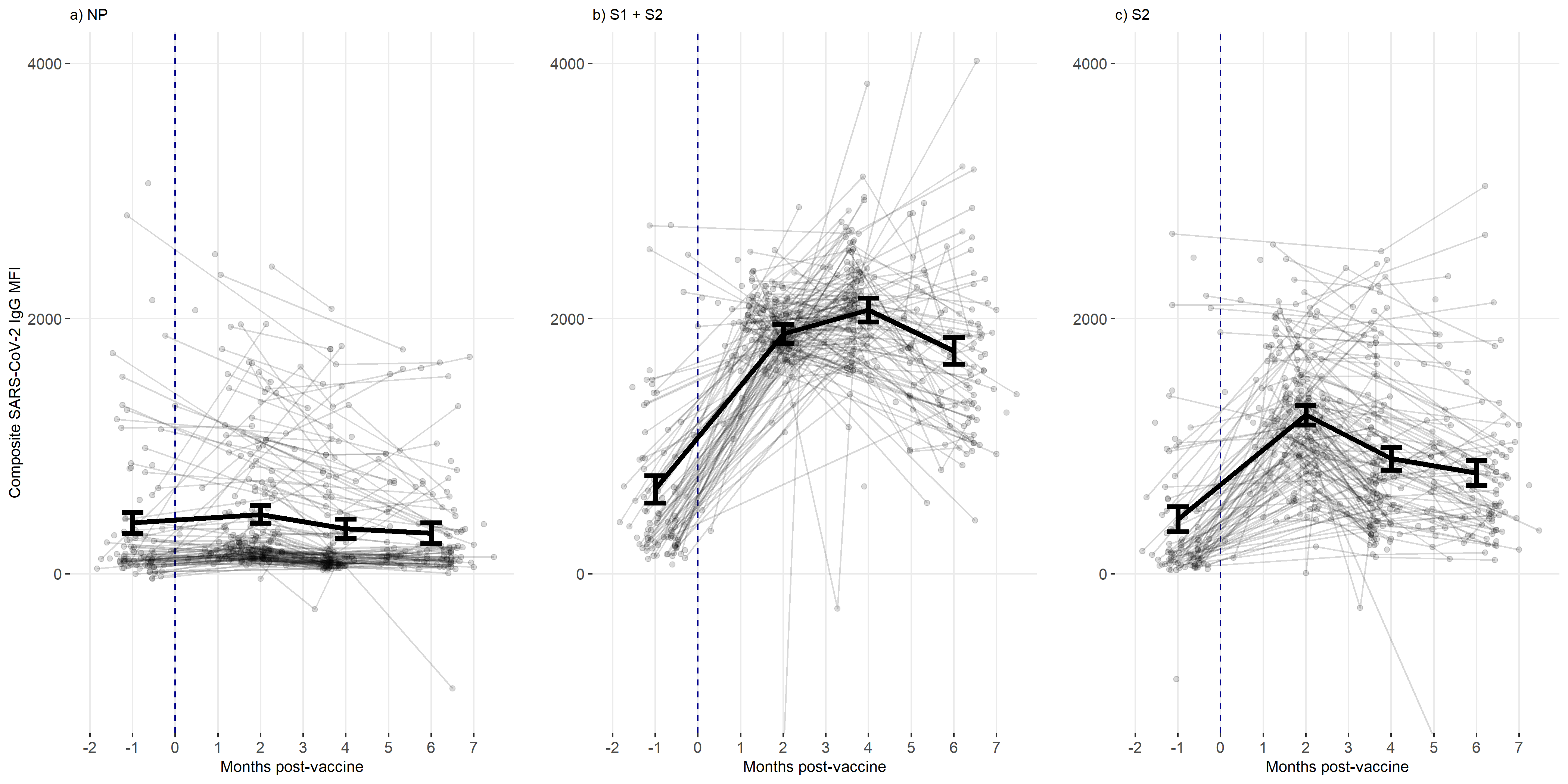

Supplement: Supplementary Figure 2 — Antigen-specific SARS-CoV-2 IgG levels over time, with background lines representing individual study participants and thick solid line representing mean antibody level at baseline, 2 months, 4 months, and 6 months with error bars representing 95% confidence intervals, for (A) NP, (B) S1+S2, and (C) S2 antigens. [file Image_2.tiff]

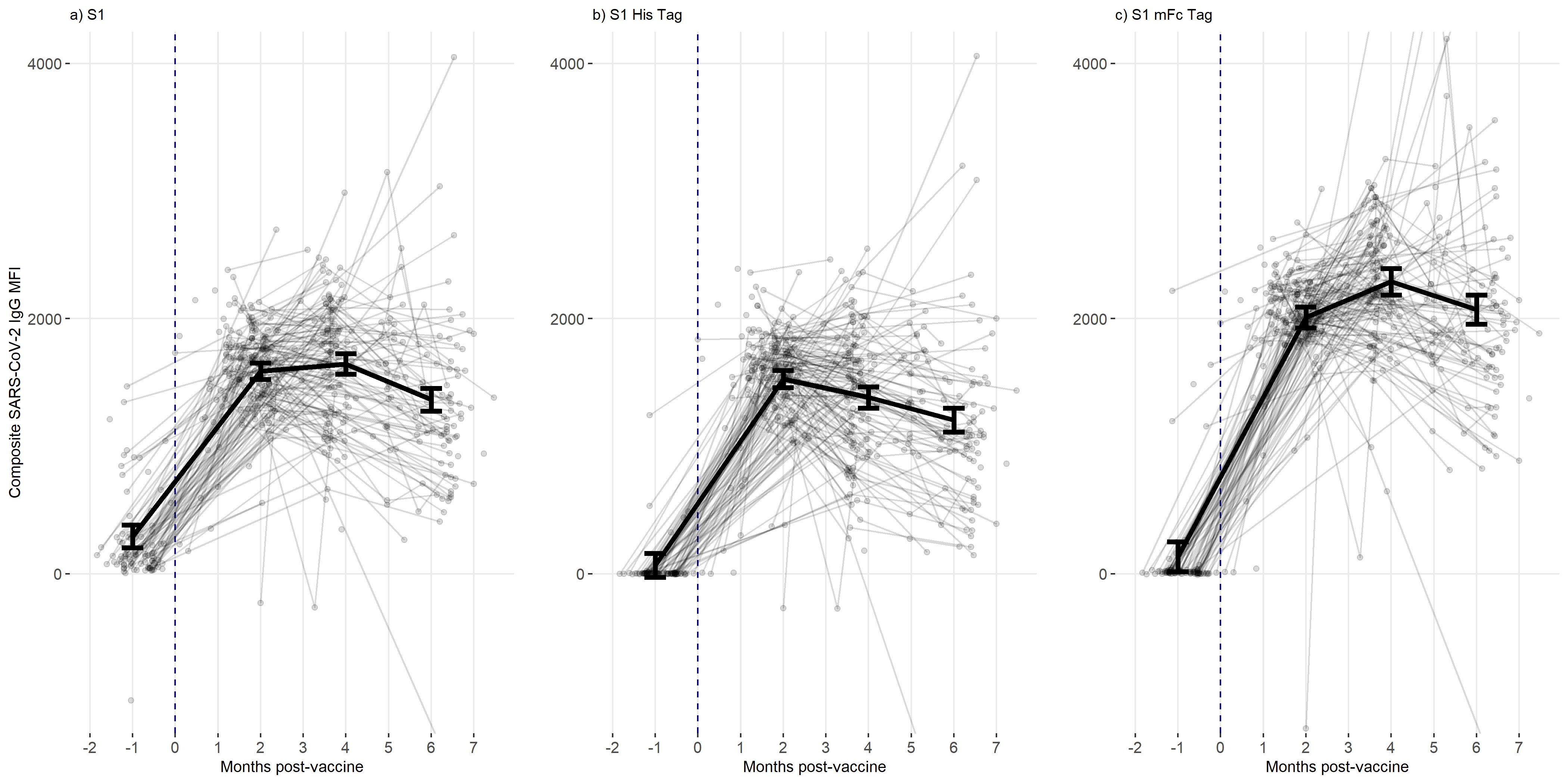

Supplement: Supplementary Figure 3 — Antigen-specific SARS-CoV-2 IgG levels over time, with background lines representing individual study participants and thick solid line representing mean antibody level at baseline, 2 months, 4 months, and 6 months with error bars representing 95% confidence intervals, for (A) S1, (B) S1 with His tag, and (C) S1 with mouse Fc tag antigens. [file Image_3.tiff]

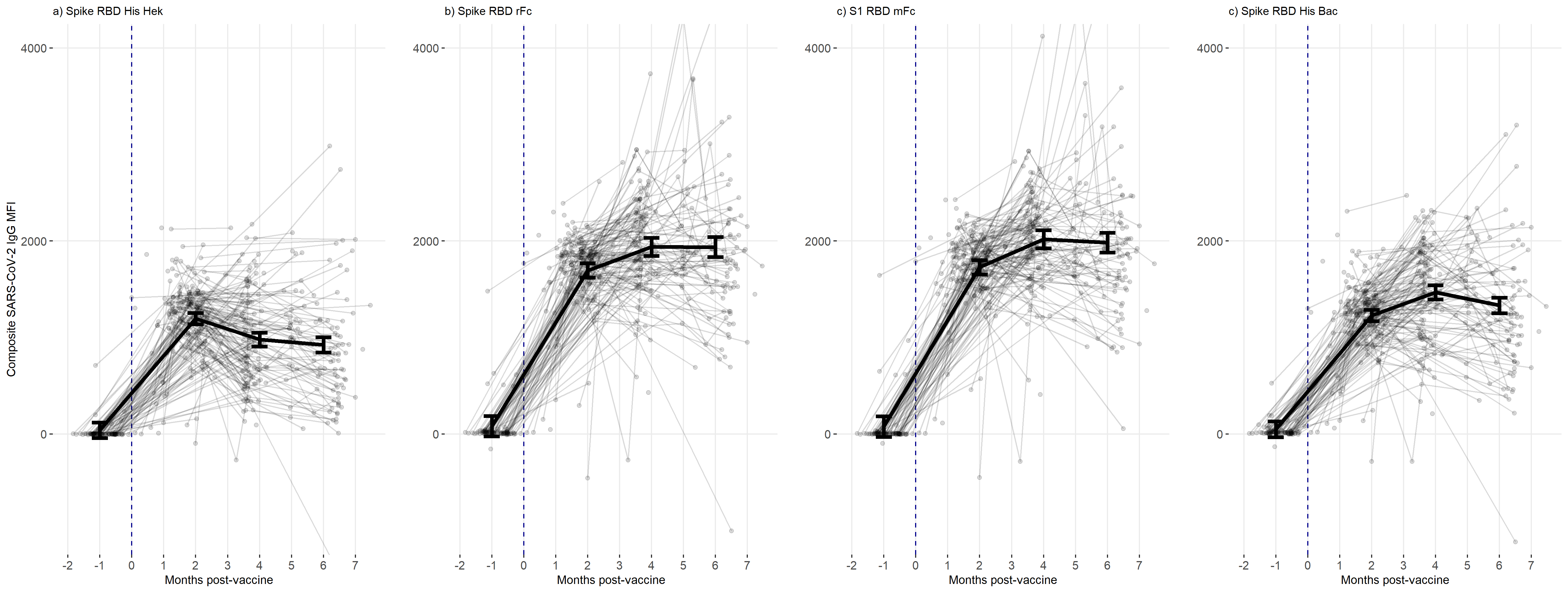

Supplement: Supplementary Figure 4 — Antigen-specific SARS-CoV-2 IgG levels over time, with background lines representing individual study participants and thick solid line representing mean antibody level at baseline, 2 months, 4 months, and 6 months with error bars representing 95% confidence intervals, for (A) RBD with His tag produced in HEK-293 cells, (B) RBD with rabbit Fc tag, (C) RBD with mouse Fc tag, and d) RBD with His tag produced in baculovirus antigens. [file Image_4.jpeg]

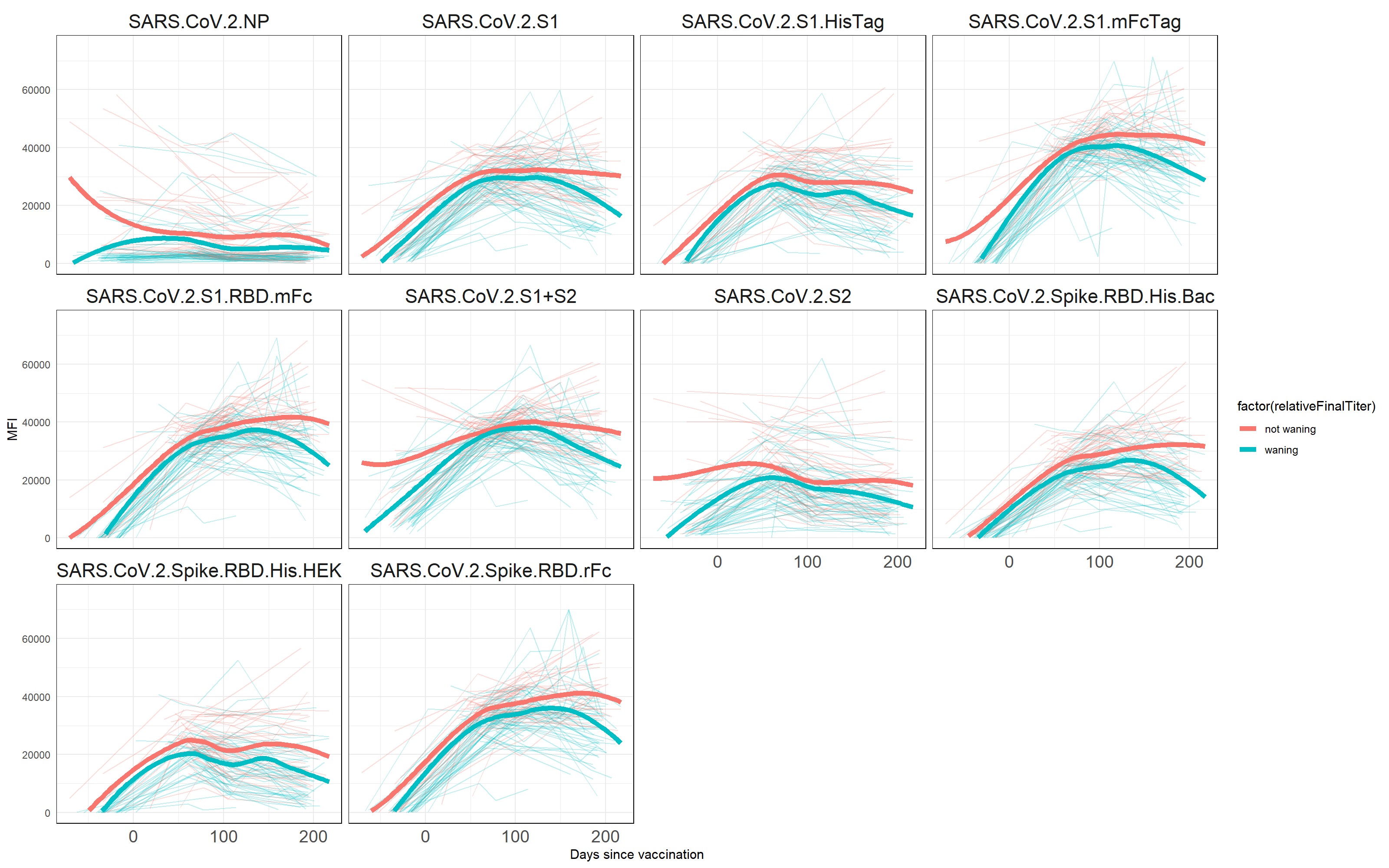

Supplement: Supplementary Figure 5 — SARS-CoV-2 reactivity after 6 months. Blue lines indicate individuals for whom reactivity did not significantly wane when compared to the last time point recorded, in red are samples for whom the reactivity has declined (P < 0.05). [file Image_5.tif]
